# Gene Polymorphisms in African Buffalo Associated with Susceptibility to Bovine Tuberculosis Infection

**DOI:** 10.1371/journal.pone.0064494

**Published:** 2013-05-15

**Authors:** Nikki le Roex, Ad P. Koets, Paul D. van Helden, Eileen G. Hoal

**Affiliations:** 1 Division of Molecular Biology and Human Genetics, DST/NRF Centre of Excellence for Biomedical Tuberculosis Research/MRC Centre for Molecular and Cellular Biology, Faculty of Health Sciences, Stellenbosch University, Cape Town, South Africa; 2 Department of Farm Animal Health, Faculty of Veterinary Medicine, Utrecht University, Utrecht, The Netherlands; 3 Department of Infectious Diseases and Immunology, Faculty of Veterinary Medicine, Utrecht University, Utrecht, The Netherlands; Institut national de la santé et de la recherche médicale - Institut Cochin, France

## Abstract

Bovine tuberculosis (BTB) is a chronic, highly infectious disease that affects humans, cattle and numerous species of wildlife. In developing countries such as South Africa, the existence of extensive wildlife-human-livestock interfaces poses a significant risk of *Mycobacterium bovis* transmission between these groups, and has far-reaching ecological, economic and public health impacts. The African buffalo (*Syncerus caffer*), acts as a maintenance host for *Mycobacterium bovis*, and maintains and transmits the disease within the buffalo and to other species. In this study we aimed to investigate genetic susceptibility of buffalo for *Mycobacterium bovis* infection. Samples from 868 African buffalo of the Cape buffalo subspecies were used in this study. SNPs (n = 69), with predicted functional consequences in genes related to the immune system, were genotyped in this buffalo population by competitive allele-specific SNP genotyping. Case-control association testing and statistical analyses identified three SNPs associated with BTB status in buffalo. These SNPs, SNP41, SNP137 and SNP144, are located in the SLC7A13, DMBT1 and IL1α genes, respectively. SNP137 remained significantly associated after permutation testing. The three genetic polymorphisms identified are located in promising candidate genes for further exploration into genetic susceptibility to BTB in buffalo and other bovids, such as the domestic cow. These polymorphisms/genes may also hold potential for marker-assisted breeding programmes, with the aim of breeding more BTB-resistant animals and herds within both the national parks and the private sector.

## Introduction

Bovine tuberculosis (BTB) is a chronic, highly infectious disease which affects humans, cattle and wildlife. BTB is caused by infection with *Mycobacterium bovis*, which is a member of the *Mycobacterium tuberculosis* complex. In developing countries such as South Africa, the existence of extensive wildlife-human-livestock interfaces poses a significant risk of transmission between these groups, and has far-reaching ecological, economic and public health impacts [1]–[3]. In addition to the impact on biodiversity and ecotourism, rural communities at these wildlife interfaces can also be severely affected, with bovine TB compromising their health, food supply and livelihoods. Many people are reliant on unpasteurised milk and meat from their livestock for food supply and high levels of HIV prevalence within these communities dramatically increases susceptibility to BTB infection [1], [3].

In the absence of a wildlife reservoir, most developed countries have effectively used test-and-cull schemes to reduce BTB prevalence in cattle to extremely low levels. This system is seldom utilised, or effective, in developing countries with a wildlife reservoir that maintains BTB within the environment [4]. In South Africa, the African buffalo (*Syncerus caffer*), acts as a maintenance host/reservoir for *M. bovis*, and thus maintains and transmits the disease within the buffalo populations and to other species [1], [5]. It is a species of enormous economic and ecological importance, due to its occurrence in large numbers in the savannah ecosystem, commercial game farming, tourism, and for hunting purposes [1]. This poses a significant obstacle to the management and control of BTB. Different BTB management strategies with regards to buffalo are implemented in the different game parks and private reserves within South Africa, and new methods of detection and control are constantly under investigation.

Studies in humans have reported multiple genetic factors and polymorphisms in a number of different genes to be associated with tuberculosis susceptibility [6]. In animal studies, the heritability of BTB resistance has been shown to be 0.48±0.09 in red deer [7], 0.18±0.04 in British dairy cattle [8] and 0.18±0.04 in Irish cattle [9], suggesting a genetic component in BTB susceptibility in these animals, which may be more pronounced in wildlife. A recent study by Sun et al. [10] investigated the role of host genetic factors in susceptibility to BTB in Chinese Holstein cattle, and found the G1596A polymorphism in the toll-like receptor 1 (TLR1) gene to be associated with BTB infection status. Finlay et al. (2012) performed a genome-wide association scan in Irish cattle and identified a genomic region on BTA 22 associated with BTB susceptibility [11]. The identification of host genetic factors involved in BTB susceptibility in African buffalo may allow for marker-assisted selection for breeding programs, as a possible long-term disease management alternative, particularly in areas currently free of BTB or with low prevalence levels.

*Mycobacterium bovis* is primarily transmitted between animals by inhalation, and to a lesser degree, ingestion [3], [12]. After inhalation, bacilli adhere to the alveolar surface of the lung and are phagocytosed by macrophages. Macrophages then process the mycobacterial antigens and present them to T-lymphocytes, which are considered essential recognition components of the immune response. Typical tuberculous lesions in buffalo occur most often in the lungs and lymph nodes, but can also be found in other, more distal sites [5]. Resistance to infection and disease could be mediated by either the innate or adaptive immune systems and therefore the genes related to either of these processes may be candidate genes for BTB susceptibility loci.

In this study, candidate polymorphisms in genes involved in the immune response were selected from the SNPs that we recently identified and validated in the African buffalo [13]. These SNPs were tested for genetic associations with BTB infection status in African buffalo, using a case-control approach.

## Materials and Methods

### Ethics

The Stellenbosch University Animal Care and Use Committee (SU ACU) deemed it unnecessary to obtain ethical clearance for this study as the blood samples used for DNA extraction were collected under the directive of SANParks and Ezemvelo KZN Wildlife for other purposes, and their use in the present study is incidental.

### Study Population

**Figure 1:**
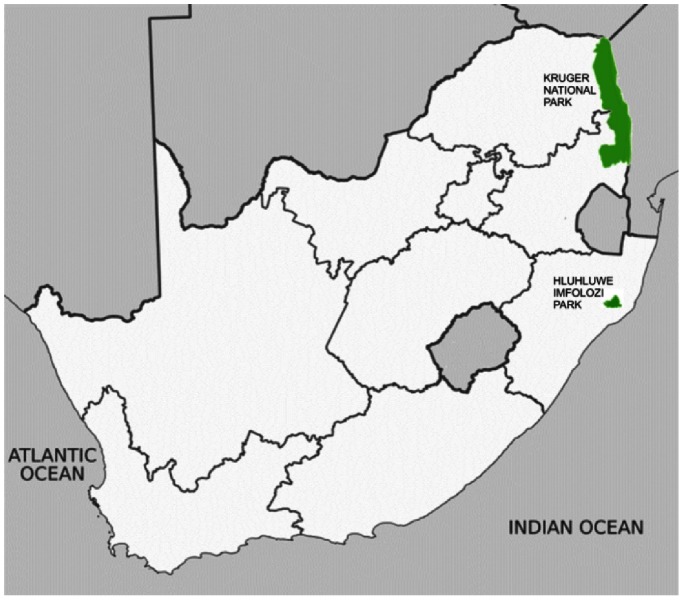
Location of the African buffalo populations in South Africa used in this study.

The DNA used in the association study was derived from 868 African buffalo (198 cases, 670 controls) of the Cape buffalo subspecies, consisting of two independent populations. The population subgroups consisted of 434 buffalo from the Kruger National Park (KNP), and 434 buffalo from Hluhluwe iMfolozi Park (HiP) (Figure 1). Both KNP and HiP have known histories of BTB in both buffalo and other wildlife species, and given the social herd structure of this species, both cases and controls were considered equally exposed. Blood samples were taken from the buffalo subpopulations during routine BTB testing operations, by qualified SANParks or Ezemvelo KZN Wildlife veterinary staff. In the KNP samples, cases and controls were defined by the positive or negative outcome of the Bovigam® ELISA assay. HiP samples were defined as cases or controls based on the results of the standard single comparative intradermal tuberculin (SCIT) test, administered following capture, with a test result considered positive when the difference in skin fold swelling between the avian and bovine inflammatory responses was greater than or equal to 2 mm. Recent publications have suggested that that a cut-off of >2 mm for positive test interpretation in cattle is most appropriate in sub-Saharan Africa [14], [15].Overall BTB prevalence in our samples in the KNP and HiP subpopulations was 22% and 23.5% respectively (own unpublished data).

### Preparation of Genomic DNA

The Kruger National Park DNA samples were extracted from blood, using a salt-chloroform extraction method [16]. KNP Samples which contained insufficient concentrations of DNA for the purposes of this study were subjected to whole genome amplification (WGA) using the Illustra Genomiphi DNA Amplification Kit (GE Healthcare Life Sciences, Piscataway, USA), which employs a strand displacement amplification technique, according to the manufacturer’s instructions. DNA was extracted from the HiP EDTA blood samples using the Qiagen DNeasy Blood and Tissue Kit (Qiagen Group®), according to the manufacturer’s instructions.

### SNP Selection and Genotyping

SNP selection was made from previously validated SNPs [13], with priority given to those with higher frequencies in the validation panel. These SNPs all had potentially functional consequences, due to their positions in the 3′UTR, 5′UTR and coding regions, in genes related to the immune system. In total, 69 SNPs were fluorescently genotyped in the full buffalo population by LGC Genomics KBioscience [17] (London, United Kingdom), using their competitive allele-specific KASP® SNP genotyping platform. The KASP® assay system relies on the discrimination power of a FRET-based homogenous form of competitive allele-specific PCR to determine the alleles at a specific locus within genomic DNA [17]. A block randomisation technique was employed to evenly distribute the samples across the 10 plates used for the genotyping in order to minimise batch effects. Variables considered included BTB infection status, location and sex. Positive controls were included on all plates to ensure consistent results. The selected SNPs and their assay IDs can be seen in Table S1.

### Statistical Analysis

Genetic differentiation between the KNP and HiP subpopulations was tested using FST values in Genepop v4.0.10 [18], [19]. Further statistical analyses were performed in Plink v1.07 (http://pngu.mgh.harvard.edu/purcell/plink/) [20]. SNPs in this study were not removed if they were out of Hardy-Weinberg Equilibrium (HWE), as due to the social structure of a buffalo herd, non-random mating is observed and thus the expectations of HWE do not hold for this data. African buffalo display a male dominance hierarchy, with a select number of dominant bulls mating with the sexually mature females, who are often related. This suggests a degree of relatedness within a herd [21]. The populations were analysed together in order to minimise the effect of any possible relatedness present in the individual herds. Quality control consisted of removing individuals with more than 10% missing data (genotyping failure) and SNPs that had a call rate of less than 90%. Pairwise linkage disequilibrium measures were calculated for all SNP pairs using correlations based on genotype allele counts, including those on different chromosomes. Allele frequencies, odds ratios (OR) and 95% confidence intervals were determined and the associated p-values were calculated using Fisher’s Exact test. A p-value <0.05 was regarded as significant. The Cochran-Mantel-Haenszel (CMH) test was used to obtain an average odds ratio and associated p-value for each SNP, adjusting for the two subpopulations. Genotypic associations with BTB infection were tested using the Cochran-Armitage trend test for the additive allele effect. The Cochran-Armitage trend test was considered the most applicable genotypic test for this study, as it does not assume HWE. Logistic regression analysis was also used to calculate odds ratios and 95% confidence intervals, assuming an additive effect of allele dosage. We controlled for subpopulation and sex by including those covariates in the logistic regression model. Permutation testing was performed using the max(T) permutation method with 5000 permutations, to obtain an empirical p-value for each SNP. SNPs found to be significantly associated with BTB status in the buffalo after correcting for the population structure were investigated further. Non-synonymous SNPs were submitted to the Ensembl Variant Effect Predictor [22] in order to establish the type and position of the amino acid changes caused.

## Results

The SNPs in this study comprised 69 loci across 64 genes, previously selected for possible functional consequence and gene location. The F_ST_ value for the genetic differentiation between the two subpopulations was 0.1345. Quality control removed 24 individuals due to missing data. No SNPs were excluded due to call rate. Because of the non-random mating structure within the buffalo herds, HWE was not tested. Two loci, SNP105 and SNP160, were identified as being in linkage disequilibrium with other SNPs (r^2^>0.5) and were excluded. Fisher’s Exact test for allelic association identified four SNPs with significantly different frequencies between cases and controls (p<0.05): SNP30, SNP41, SNP137 and SNP144. After adjusting for differences due to the subpopulations using the CMH test, three of the four previously identified SNPs remained significantly associated. Following correction for false positives using the max(T) permutation method, SNP137 was identified as significantly associated with BTB status. Associated SNPs in the allelic association and CMH tests, and the accompanying odds ratios and p values, can be seen in Table 1. SNP30 and SNP144 are located in the 3′UTRs of the complement component C7 gene and the interleukin 1-alpha (IL1α) gene, respectively. SNP41 and SNP137 are located in the coding regions of the solute carrier family 7 (anionic amino acid transporter), member 13 (SLC7A13) gene and Deleted in Malignant Brain Tumour (DMBT1) genes, respectively. All SNPs, allele frequencies, odds ratios and p values for the CMH test can be seen in Table S2.

**Table 1:** Allele frequencies, odds ratios and p-values of four significant SNPs.

SNP	Allele	Fcase	Fcont	OR (95% CI)	P	CMH OR (95% CI)	CMH P	P_emp_
SNP30	G	0.2251	0.2764	0.7606 (0.581–0.995)	0.048	0.7651 (0.584–1.002)	0.05136	0.9732
SNP41	C	0.3474	0.2851	1.335 (1.047–1.702)	0.0218	1.377 (1.075–1.764)	0.01157	0.5591
SNP137	G	0.4229	0.3323	1.472 (1.163–1.863)	0.0014	1.608 (1.243–2.079)	0.00028	0.0238
SNP144	T	0.3534	0.2941	1.312 (1.03–1.67)	0.0318	1.421 (1.095–1.843)	0.00793	0.4313

P_emp_: Empirical p-value obtained by max(T) permutation.

The Cochran-Armitage trend test for genotypic association identified the same four SNPs (SNP30, SNP41, SNP137 and SNP144) as significant. No significant associations remained after correction using max(T) permutation.

Logistic regression analysis incorporating both sex and subpopulation as covariates identified SNP41, SNP137 and SNP144 as significantly associated with BTB status. Logistic regression analysis incorporating only subpopulation as a covariate produced the same result, and sex was not identified as a significant factor in this study (data not shown). After correction using max(T) permutation, SNP137 remained significantly associated. We note that the SNP137 was also significantly associated in the allelic, genotypic and logistic regression tests in both the HiP and KNP populations when analysed separately (data not shown). The significant logistic regression model results, odds ratios and p-values can be seen in Table 2, and the logistic regression results for all SNPs in Table S3.

**Table 2:** Logistic regression model with significant SNPs, odds ratios and p-values.

SNP	Allele	Test	OR (95% CI)	P	P_perm_
SNP41	C	ADD	1.349 (1.06–1.715)	0.01474*	0.6265
SNP41	C	COV1^a^	1.222 (0.8746–1.708)	0.2397	
SNP137	G	ADD	1.574 (1.221–2.029)	0.000461*	0.02599*
SNP137	G	COV1	1.47 (1–2.16)	0.04982*	
SNP144	T	ADD	1.435 (1.101–1.87)	0.007569*	0.3893
SNP144	T	COV1	1.396 (0.9605–2.029)	0.08032	

a COV1 refers to the population subgroup.

* denotes statistical significance, p<0.05.

The amino acid changes caused by SNP41 and SNP137, as well as their positions in their respective proteins, were identified by the Ensemble Variant Effect Predictor (Table 3). Although both the DMBT1 and SLC7A13 proteins are uncharacterised in the cow, SNP137 was predicted to occur in a CUB domain within the DMBT1 protein. CUB domains are evolutionarily conserved protein domains found commonly in extracellular and plasma-membrane associated proteins [23].

**Table 3:** Ensembl Variant Effect Predictor predictions of amino acid changes and positions.

SNP	Location	Allele	Ensembl Gene ID	Consequence	Position in cDNA	Position in CDS^a^	Position in protein	Amino acid change
41	14:79862771	C	ENSBTAG00000040461	missense	1444	1330	444	I/V
137	26:42797281	G	ENSBTAG00000022715	missense	3350	3350	1117	H/R

a CDS refers to the coding sequence.

## Discussion

To our knowledge, this is the first report of genetic susceptibility to bovine TB in the African buffalo. In other bovids, specifically the domestic cow, very few loci have been identified, apart from microsatellite loci [24], TLR1 [10] and a genomic region on BTA 22 [11]. In this study, we investigated 69 SNPs located in genes relating to the immune system for association with BTB infection status in African buffalo, using a large sample set of 868 buffalo samples from two independent populations, the Kruger National Park and Hluhluwe iMfolozi Park. Prior to correction for the population subgroups, four SNPs were identified as associated with BTB status – SNP30, SNP41, SNP137 and SNP144. The F_ST_ value of 0.13 suggests that there is a degree of differentiation between the buffalo populations, which necessitates controlling for. After correction for population subgroups using the Cochran-Mantel-Haenszel test and logistic regression analysis, three SNPs remained significantly associated: SNP41, SNP137 and SNP144.

SNP41 occurs in the SLC7A13 gene, and is predicted to cause a non-synonymous amino acid substitution at position 444 in this protein, from isoleucine to valine. The SLC7A13 gene forms part of the family of heteromeric amino acid transporters (HATs). HATs are comprised of a heavy and a light chain/subunit, and the SLC7A13 gene encodes one of the light subunits [25], [26]. The light subunits are highly hydrophobic and are suggested to have a 12 transmembrane-domain topology [25]. While SLC7A13 is relatively uncharacterised, other transporters, such as SLC11A1 (NRAMP1), have been associated with TB infection in humans [27], [28].

SNP137 is located in the coding region of the Deleted in Malignant Brain Tumour-1 (DMBT1) gene. This SNP is predicted to cause a substitution at position 1117 of the protein, from histidine to arginine. The DMBT1 gene produces a number of different protein isoforms, all of which belong to the scavenger receptor cysteine-rich (SRCR) superfamily of proteins. SRCR proteins are highly conserved throughout the metazoa, and contain two CUB domains and one zona pellucida domain [29]. The glycoprotein isoforms have been shown to have a range of functions that include the binding of both gram-positive and gram-negative bacteria, binding to viral proteins, and binding to host surfactant proteins A and D, among others, which are collectins that play an important role in the innate immune response [30], [31]. Both DMBT1 and the mouse homolog of this protein (CRP-ductin) are regarded as pattern recognition receptors (PRRs), which are a well-established part of the human host defence system against invading organisms such as *M. tuberculosis*. PRRs recognize conserved structures in bacterial cell walls, yeast cell walls, and bacterial DNA that are vital to the survival of the invading organism [29], [32]. Many members of the SRCR superfamily have been shown to be located on immune cells such as B-lymphocytes, T-lymphocytes and macrophages, and the role of the DMBT1 proteins in the innate immune response has been well established [30]. While evidence is lacking for the bovine protein, the conserved nature of these proteins suggests a similar function in the domestic cow.

SNP144 is found in the 3′ untranslated region (UTR) of the Interleukin 1 alpha (IL1α) gene. The IL1α protein belongs to the IL1 family of pro-inflammatory cytokines, and plays a role in the activation of T-lymphocytes, the recruitment of leucocytes to sites of inflammation, and mediating local and systemic inflammation, and is expressed predominantly by macrophages and neutrophils [33], [34]. A study by Bellamy et al. [35] found a significant association between the IL1 gene cluster and *M. tuberculosis* in humans, although this was lost for IL1α after Bonferroni correction.

While the results of this study are promising, there were some limitations, most notably the difficulties of working with a non-model organism without an assembled genome or well-established population data. Because our buffalo herd data falls somewhere between that of population data and family data, and the degree of relatedness among the cases and controls prevented the investigation of SNPxSNP interactions. The second limitation faced was the different diagnostic methods used for the two population groups. The Bovigam® ELISA assay was used to diagnose BTB infection in the Kruger National Park buffalo, and the SCIT test was used in Hluhluwe iMfolozi Park, but since both of these tests are based on a cell-mediated immune response and therefore expected to detect animals at a similar stage of infection [36], [37], this was not considered problematic in this study.

In conclusion, our study has identified three genetic polymorphisms, in the SLC7A13, IL1α and DMBT1 genes, that are associated with bovine TB infection status in the African buffalo. The association of the three polymorphisms listed above were robust to correction for buffalo population, and one remained significant after permutation testing. The large number of individuals used in this study, the randomisation procedure and statistical controls employed, and the plausibility of the genes identified suggest that these polymorphisms are located in excellent candidate genes for further exploration into genetic susceptibility to BTB in both the buffalo and other bovids, such as the domestic cow. These genes may also have potential for marker-assisted breeding programmes, with the aim of breeding more BTB resistant animals and herds within both the national parks and the private sector.

## Supporting Information

Table S1 **SNP identifiers, Assay IDs, UMD3 positions, Ensembl gene ID and names, consequences.**
(TXT)

Table S2 **All allele frequencies, odds ratios and p-values for Cochran-Mantel-Haenszel test.**
(TXT)

Table S3 **Logistic regression odds ratios and p-values for all SNPs.**
(TXT)

## References

[1] Michel AL, Coetzee ML, Keet DF, Maré L, Warren R, et al (2009) Molecular epidemiology of Mycobacterium bovis isolates from free-ranging wildlife in South African game reserves. Veterinary Microbiology 133: 335–343 doi:16/j.vetmic.2008.07.023. 18786785 10.1016/j.vetmic.2008.07.023

[2] Michel AL, Bengis RG, Keet DF, Hofmeyr M, Klerk LM, et al (2006) Wildlife tuberculosis in South African conservation areas: implications and challenges. VetMicrobiol 112: 91–100. 10.1016/j.vetmic.2005.11.035 16343819

[3] Ayele WY, Neill SD, Zinsstag J, Weiss MG, Pavlik I (2004) Bovine tuberculosis: an old disease but a new threat to Africa. IntJTubercLung Dis 8: 924–937. 15305473

[4] Caron A, Cross PC, Du Toit JT (2003) Ecological implications of bovine tuberculosis in African buffalo herds. Ecological Applications 13: 1338–1345.

[5] Renwick AR, White PCL, Bengis RG (2007) Bovine tuberculosis in southern African wildlife: a multi-species host-pathogen system. Epidemiol Infect 135: 529–540 doi:10.1017/S0950268806007205. 16959052 10.1017/S0950268806007205 PMC2870607

[6] Möller M, Hoal EG (2010) Current findings, challenges and novel approaches in human genetic susceptibility to tuberculosis. Tuberculosis (Edinb) 90: 71–83 doi:10.1016/j.tube.2010.02.002. 20206579 10.1016/j.tube.2010.02.002

[7] Mackintosh CG, Qureshi T, Waldrup K, Labes RE, Dodds KG, et al (2000) Genetic resistance to experimental infection with Mycobacterium bovis in red deer (Cervus elaphus). InfectImmun 68: 1620–1625. 10.1128/iai.68.3.1620-1625.2000 PMC97322 10678981

[8] Brotherstone S, White IMS, Coffey M, Downs SH, Mitchell AP, et al (2010) Evidence of genetic resistance of cattle to infection with Mycobacterium bovis. J Dairy Sci 93: 1234–1242 doi:10.3168/jds.2009-2609. 20172243 10.3168/jds.2009-2609

[9] Bermingham ML, More SJ, Good M, Cromie AR, Higgins IM, et al (2009) Genetics of tuberculosis in Irish Holstein-Friesian dairy herds. JDairy Sci 92: 3447–3456. 19528623 10.3168/jds.2008-1848

[10] Sun L, Song Y, Riaz H, Yang H, Hua G, et al (2012) Polymorphisms in toll-like receptor 1 and 9 genes and their association with tuberculosis susceptibility in Chinese Holstein cattle. Vet Immunol Immunopathol 147: 195–201 doi:10.1016/j.vetimm.2012.04.016. 22572235 10.1016/j.vetimm.2012.04.016

[11] Finlay EK, Berry DP, Wickham B, Gormley EP, Bradley DG (2012) A genome wide association scan of bovine tuberculosis susceptibility in Holstein-Friesian dairy cattle. PLoS ONE 7: e30545 doi:10.1371/journal.pone.0030545. 22355315 10.1371/journal.pone.0030545 PMC3280253

[12] Hope JC, Villarreal-Ramos B (2008) Bovine TB and the development of new vaccines. Comp ImmunolMicrobiolInfectDis 31: 77–100. 10.1016/j.cimid.2007.07.003 17764740

[13] Le Roex N, Noyes H, Brass A, Bradley DG, Kemp SJ, et al (2012) Novel SNP Discovery in African Buffalo, Syncerus caffer, Using High-Throughput Sequencing. PLoS ONE 7: e48792 doi:10.1371/journal.pone.0048792. 23144973 10.1371/journal.pone.0048792 PMC3492240

[14] Ameni G, Hewinson G, Aseffa A, Young D, Vordermeier M (2008) Appraisal of Interpretation Criteria for the Comparative Intradermal Tuberculin Test for Diagnosis of Tuberculosis in Cattle in Central Ethiopia. Clinical and Vaccine Immunology 15: 1272–1276 doi:10.1128/CVI.00114-08. 18495847 10.1128/CVI.00114-08 PMC2519295

[15] Ngandolo BN, Muller B, guimbaye-Djaibe C, Schiller I, Marg-Haufe B, et al (2009) Comparative assessment of fluorescence polarization and tuberculin skin testing for the diagnosis of bovine tuberculosis in Chadian cattle. PrevVetMed 89: 81–89. 10.1016/j.prevetmed.2009.02.003 19269049

[16] Miller SA, Dykes DD, Polesky HF (1988) A simple salting out procedure for extracting DNA from human nucleated cells. Nucleic Acids Res 16: 1215. 3344216 10.1093/nar/16.3.1215 PMC334765

[17] LGC Genomics KBioscience (n.d.). Available:http://www.lgcgenomics.com/. Accessed 2013 Apr 22.

[18] Raymond M, Rousset F (1995) GENEPOP (Version 1.2): Population Genetics Software for Exact Tests and Ecumenicism. J Hered 86: 248–249.

[19] Rousset F (2008) genepop’007: a complete re-implementation of the genepop software for Windows and Linux. Molecular Ecology Resources 8: 103–106 doi:10.1111/j.1471-8286.2007.01931.x. 21585727 10.1111/j.1471-8286.2007.01931.x

[20] Purcell S, Neale B, Todd-Brown K, Thomas L, Ferreira MAR, et al (2007) PLINK: A Tool Set for Whole-Genome Association and Population-Based Linkage Analyses. American Journal of Human Genetics 81: 559. 17701901 10.1086/519795 PMC1950838

[21] Simonsen BT, Siegismund HR, Arctander P (1998) Population structure of African buffalo inferred from mtDNA sequences and microsatellite loci: high variation but low differentiation. MolEcol 7: 225–237. 10.1046/j.1365-294x.1998.00343.x 9532761

[22] McLaren W, Pritchard B, Rios D, Chen Y, Flicek P, et al (2010) Deriving the consequences of genomic variants with the Ensembl API and SNP Effect Predictor. Bioinformatics 26: 2069–2070 doi:10.1093/bioinformatics/btq330. 20562413 10.1093/bioinformatics/btq330 PMC2916720

[23] Blanc G, Font B, Eichenberger D, Moreau C, Ricard-Blum S, et al (2007) Insights into how CUB domains can exert specific functions while sharing a common fold: conserved and specific features of the CUB1 domain contribute to the molecular basis of procollagen C-proteinase enhancer-1 activity. J Biol Chem 282: 16924–16933 doi:10.1074/jbc.M701610200. 17446170 10.1074/jbc.M701610200

[24] Driscoll EE, Hoffman JI, Green LE, Medley GF, Amos W (2011) A preliminary study of genetic factors that influence susceptibility to bovine tuberculosis in the British cattle herd. PLoS ONE 6: e18806 doi:10.1371/journal.pone.0018806. 21533277 10.1371/journal.pone.0018806 PMC3075270

[25] Palacín M, Nunes V, Font-Llitjós M, Jiménez-Vidal M, Fort J, et al (2005) The genetics of heteromeric amino acid transporters. Physiology (Bethesda) 20: 112–124 doi:10.1152/physiol.00051.2004. 15772300 10.1152/physiol.00051.2004

[26] Verrey F, Closs EI, Wagner CA, Palacin M, Endou H, et al (2004) CATs and HATs: the SLC7 family of amino acid transporters. Pflugers Arch 447: 532–542 doi:10.1007/s00424-003-1086-z. 14770310 10.1007/s00424-003-1086-z

[27] Velez DR, Hulme WF, Myers JL, Stryjewski ME, Abbate E, et al (2009) Association of SLC11A1 with tuberculosis and interactions with NOS2A and TLR2 in African-Americans and Caucasians. Int J Tuberc Lung Dis 13: 1068–1076. 19723394 PMC2902362

[28] Hoal EG, Lewis LA, Jamieson SE, Tanzer F, Rossouw M, et al (2004) SLC11A1 (NRAMP1) but not SLC11A2 (NRAMP2) polymorphisms are associated with susceptibility to tuberculosis in a high-incidence community in South Africa. Int J Tuberc Lung Dis 8: 1464–1471. 15636493

[29] Ligtenberg AJM, Karlsson NG, Veerman ECI (2010) Deleted in Malignant Brain Tumors-1 Protein (DMBT1): A Pattern Recognition Receptor with Multiple Binding Sites. Int J Mol Sci 11: 5212–5233 doi:10.3390/ijms1112521. 21614203 10.3390/ijms1112521 PMC3100851

[30] Madsen J, Mollenhauer J, Holmskov U (2010) Review: Gp-340/DMBT1 in mucosal innate immunity. Innate Immun 16: 160–167 doi:10.1177/1753425910368447. 20418254 10.1177/1753425910368447

[31] Palaniyar N (2010) Antibody equivalent molecules of the innate immune system: parallels between innate and adaptive immune proteins. Innate Immun 16: 131–137 doi:10.1177/1753425910370498. 20529970 10.1177/1753425910370498

[32] Kleinnijenhuis J, Oosting M, Joosten LAB, Netea MG, Van Crevel R (2011) Innate immune recognition of Mycobacterium tuberculosis. Clin Dev Immunol 2011: 405310 doi:10.1155/2011/405310. 21603213 10.1155/2011/405310 PMC3095423

[33] Schmidt DR, Kao WJ (2007) The interrelated role of fibronectin and interleukin-1 in biomaterial-modulated macrophage function. Biomaterials 28: 371–382 doi:10.1016/j.biomaterials.2006.08.041. 16978691 10.1016/j.biomaterials.2006.08.041

[34] Beltan E, Horgen L, Rastogi N (2000) Secretion of cytokines by human macrophages upon infection by pathogenic and non-pathogenic mycobacteria. Microb Pathog 28: 313–318 doi:10.1006/mpat.1999.0345. 10799281 10.1006/mpat.1999.0345

[35] Bellamy R, Ruwende C, Corrah T, McAdam KPWJ, Whittle HC, et al (1998) Assessment of the interleukin 1 gene cluster and other candidate gene polymorphisms in host susceptibility to tuberculosis. Tubercle and Lung Disease 79: 83–89 doi:10.1054/tuld.1998.0009. 10645445 10.1054/tuld.1998.0009

[36] Vordermeier M, Goodchild A, Clifton-Hadley R, De la Rua R (2004) The interferon-gamma field trial: background, principles and progress. Vet Rec 155: 37–38. 15285281

[37] De la Rua-Domenech, Goodchild AT, Vordermeier HM, Hewinson RG, Christiansen KH, et al (2006) Ante mortem diagnosis of tuberculosis in cattle: a review of the tuberculin tests, gamma-interferon assay and other ancillary diagnostic techniques. ResVetSci 81: 190–210. 10.1016/j.rvsc.2005.11.005 16513150

